# Diclofenac Potassium in Acute Postoperative Pain and Dysmenorrhoea: Results from Comprehensive Clinical Trial Reports

**DOI:** 10.1155/2018/9493413

**Published:** 2018-01-17

**Authors:** R. Andrew Moore, Sheena Derry

**Affiliations:** Pain Research, Nuffield Division of Anaesthetics, University of Oxford, The Churchill, OX3 7LE Oxford, UK

## Abstract

We compared the efficacy of diclofenac potassium in unpublished clinical study reports (CSRs) and published reports to examine publication bias, industry bias, and comprehensiveness. Novartis provided CSRs of randomised double-blind trials of diclofenac potassium involving postoperative patients following third molar extraction (3 trials, *n*=519), gynaecological surgery (3 trials, *n*=679), and dysmenorrhoea (2 trials, *n*=711) conducted in 1988–1990. Searches identified published reports of 6 trials. Information from 599/1909 patients was not published; trials with 846/1909 patients were published in a defunct journal. Greater methodological information in CSRs contributed to lesser risk of bias than published trials. Numbers needed to treat (NNT) from CSRs for all six postoperative trials for at least 50% of maximum pain relief over 6 h were 2.2 (95% confidence interval, 1.9–2.6) and 2.1 (1.8–2.4) for 50 and 100 mg diclofenac potassium, respectively. A Cochrane review of published trial data reported NNTs of 2.1 and 1.9, and one comprehensive analysis reported NNTs of 2.2 and 2.1, respectively. All analyses had similar results for patients remedicating within 8 h. No data from dysmenorrhoea CSRs appeared in a Cochrane review. CSRs provide useful information and increase confidence. Stable efficacy estimates with standard study designs reduce the need for updating reviews.

## 1. Introduction

Systematic reviews are meant to include all relevant information, and considerable effort goes into ensuring comprehensiveness. At the same time, researchers need to be alert to the dangers of including duplicate reports of trial(s) as this can inflate treatment effects [1]. There is growing interest in assessment of efficacy and harm using company clinical study reports (CSRs) because of limits in the amount of information in articles published in the medical literature [2–4]. A number of reviews have evaluated CSRs in chronic pain [3–5], and individual patient-level analyses have been used in chronic pain [6, 7], acute postoperative pain [8–10], and dysmenorrhoea [11].

Nonsteroidal anti-inflammatory drugs (NSAIDs) such as ibuprofen and diclofenac have been available for 40 years or more. Fast-acting oral formulations produce more rapid absorption and higher, earlier, concentrations in blood, giving greater efficacy for the same dose [12–14]. Recent analyses suggest that historical advice to take NSAIDs with food can delay absorption and may reduce efficacy [12, 15].

Both developments are relevant to diclofenac, which is available in two salt formulations. Diclofenac sodium is usually available as enteric-coated tablets that resist dissolution in the low pH of the gastric environment, with release of drug in the duodenum [16]. This tends to produce slow absorption from the duodenum into blood, and this formulation is designed for chronic painful conditions. Diclofenac potassium is formulated for rapid release and absorption in the stomach, with peak plasma concentrations achieved by about 45 minutes [17], making it suitable for acute painful conditions. These two formulations produce very different results in acute pain trials: 50 mg diclofenac potassium had an NNT of 2.1 (95% confidence interval 1.9 to 2.5), whilst that for 50 mg of diclofenac sodium was 6.6 (4.4 to 17) [13]. Choice of formulation can be as important as the drug itself for diclofenac, and also ibuprofen [14].

We originally aimed to perform a systematic review of CSRs of diclofenac potassium using what was thought to be unpublished material and to compare the results of diclofenac potassium efficacy from unpublished CSRs with that from published reports to examine potential effects of publication bias, industry bias, and comprehensiveness. In doing so, we discovered some unusual, and we think new, insights relating to understanding how comprehensiveness impacts on estimates of efficacy.

## 2. Methods

This study developed out of a review of the cardiovascular safety of NSAIDs triggered by several regulatory authorities in 2013-2014. Novartis prepared a comprehensive list of all clinical trials of diclofenac as a part of a benefit-risk evaluation of the compound. Initial research based on that comprehensive list involved analyses relating to diclofenac (mainly as the sodium salt) in chronic pain [18, 19]. One of us (RAM) acted as an advisor and a partner.

The comprehensive list also identified a number of studies of diclofenac potassium in acute pain, performed in the late 1980s and early 1990s. At the time, these were thought to be unpublished; a project plan was developed by RAM for a review of these “unpublished” trials based on the clinical study reports, as individual patient-level data were not available. We compared study characteristics of CSRs with published reports as part of due diligence to protect against including duplicated data, and so the direction of the study changed to examining the impact of available data on estimates of efficacy.

### 2.1. Searching for Randomised Trials and Reviews

Randomised, double-blind trials of diclofenac potassium in acute postoperative pain or dysmenorrhoea sponsored by Novartis Pharma AG or its predecessor companies were retrieved from the Novartis comprehensive database. We sought clinical study reports or (ideally) study data available at the level of the individual patient. Trials had to have been concluded by the end of 2013 to be eligible, as analysis and compilation of a CSR takes time.

Electronic searches were carried out for systematic reviews and meta-analyses of diclofenac potassium in acute postoperative pain and dysmenorrhoea, using PubMed with the “Review” filter both on and off. The date of the last electronic search was December 2015. There was no restriction as to language or date in our searches.

We also sought relevant published randomised trials identified from the Cochrane reviews of diclofenac in acute postoperative pain [13] and dysmenorrhoea [20]. We ran additional searches using search strategies in those reviews, and for any older data, using our in-house database of pre-1994 randomised trials created by hand-searching journals from their inception [21].

### 2.2. Inclusion and Exclusion Criteria for Randomised Trials and Reviews

We included randomised double-blind trials of single dose oral diclofenac potassium compared with placebo for the treatment of moderate to severe postoperative pain or dysmenorrhoea in adults, with at least 10 participants randomly allocated to each treatment group. Multiple-dose studies were included if appropriate data from the first dose were available; crossover studies were included if information from the first arm was presented separately. Postpartum pain studies were included if the pain investigated was due to episiotomy or caesarean section irrespective of the presence of uterine cramps; studies investigating pain due to uterine cramps alone were not included. Participants had to be adults (15 years or older is the usual definition in third molar extraction studies) with established postoperative pain of moderate to severe intensity following day surgery or inpatient surgery, or moderate or severe pain from dysmenorrhoea. A visual analogue scale (VAS) pain intensity of greater than 30 mm was considered to be pain of at least moderate intensity [22].

We excluded case reports and clinical observations, studies of experimental pain, studies where pain relief was assessed only by clinician, nurse, or other carers and not reported by the patient themselves, studies of less than four hours' duration, or studies that failed to present data over four to six hours after dose.

Any systematic review or meta-analysis was eligible for inclusion. For the Cochrane reviews, only the most recently updated version was selected.

### 2.3. Risk of Bias Assessment

We assessed the potential risk of bias in all the included randomised trials. The Oxford Quality Score was the basis for study inclusion, limiting inclusion to studies that were randomised and double-blind as a minimum [23].

We also completed a “Risk of bias” table using methods adapted from Cochrane. The authors independently assessed risk of bias for each study using the criteria outlined in the *Cochrane Handbook for Systematic Reviews of Interventions*, resolving any disagreements by discussion [24]. The following were assessed for each study:

1. Random sequence generation (checking for possible selection bias): we assessed the method used to generate the allocation sequence as low risk of bias (any truly random process: random number table; computer random number generator) and unclear risk of bias (method used to generate sequence not clearly stated). We excluded studies using a nonrandom process, which were therefore at high risk of bias (odd or even date of birth; hospital or clinic record number).
2. Allocation concealment (checking for possible selection bias): we assessed the method used to conceal allocation of the intervention as low risk of bias (telephone or central randomisation; consecutively numbered sealed opaque envelopes) and unclear risk of bias (method not clearly stated). We excluded studies that did not conceal allocation, which were therefore at high risk of bias (open list).
3. Blinding of participants, study personnel, and outcome assessment (checking for possible performance and detection bias): we assessed the methods used to blind study participants and personnel from knowledge of which intervention a participant received as low risk of bias (study stated that it was blinded and described the method used to achieve blinding: identical tablets; matched in appearance and smell) and unclear risk of bias (study stated that it was blinded but did not provide an adequate description of how blinding was achieved). We excluded studies that were not double-blind and therefore at high risk of bias.
4. Size (checking for possible biases confounded by small size): small studies have been shown to overestimate treatment effects, probably because the conduct of small studies is more likely to be less rigorous, allowing critical criteria to be compromised [25–27]. We considered studies to be at low risk of bias if they had 200 participants or more, at unclear risk if they had 50 to 199 participants, and at high risk if they had fewer than 50 participants.

A potential bias in trials involves missing data and use of imputation using last observation carried forward when a patient requests rescue medication. This does not affect results for up to six hours after taking study medication in acute pain trials [8, 28].

### 2.4. Outcome Measures

The primary pain outcome was that of participants achieving at least 50% pain relief over four to six hours after study drug administration. This outcome has been used for over 20 years, validated several times as sensitively discriminating between analgesics of different efficacy, and is of value in postoperative pain [10, 29] and women with dysmenorrhoea [11].

For each study, we converted the mean total pain relief (TOTPAR) and summed pain intensity difference (SPID), visual analogue scale (VAS) TOTPAR, or VAS SPID values for the active and placebo groups to %maxTOTPAR or %maxSPID by division by the calculated maximum value [30]. We then calculated the proportion of participants in each treatment group who achieved at least 50% maxTOTPAR using verified equations [31–33]. We converted these proportions into the number of participants achieving at least 50% maxTOTPAR by multiplying by the total number of participants in the treatment group. Though these methods were developed for acute postoperative pain and have been thoroughly validated in various postoperative pain models, they have not been validated for dysmenorrhoea trials. However, as the dysmenorrhoea trials here used exactly the same methods and scales, we felt justified in attempting a similar approach, especially as the results for pain relief could be assessed against global impression and remedication outcomes.

We accepted the following pain measures for the calculation of TOTPAR or SPID, in order of priority:

- Five-point categorical pain relief (PR) scales with comparable wording to “none, slight, moderate, good, or complete”
- Four-point categorical pain intensity (PI) scales with comparable wording to “none, mild, moderate, or severe”
- VAS for pain relief
- VAS for pain intensity

Other outcomes looked for were as follows:

- Patients reporting a global evaluation as “very good” or “excellent” on a five-point categorical global scale with the wording “poor, fair, good, very good, or excellent”
- Patients using rescue medication within a particular time [34]
- Patients with any adverse event, any serious adverse event (as reported in the study), and withdrawal due to an adverse event

### 2.5. Statistical Analysis

For efficacy analyses, we used a number of participants in each treatment group who were randomised, received medication, and provided at least one postbaseline assessment. For safety analyses, we used a number of participants randomised to each treatment group who took the study medication. Pooled analyses were performed by pain condition and diclofenac dose using data in the CSRs only, for comparison with results from the Cochrane reviews. We also performed a pooled analysis for all available diclofenac data in all postoperative studies for 50 mg and 100 mg doses of diclofenac potassium and irrespective of formulation, for both data from the CSRs and for all available identified studies. We used CSRs rather than published trial data when both were available.

Risk ratio (or relative risk, RR) was used to establish statistical difference, and numbers needed to treat for one additional beneficial outcome (NNT) and pooled percentages as absolute measures of benefit or harm. The following terms were used to describe adverse outcomes in terms of harm or prevention of harm:

- When significantly fewer adverse outcomes occurred with treatment than with control (placebo or active), we used the term the number needed to treat to prevent one harmful event (NNTp).
- When significantly more adverse outcomes occurred with treatment compared with control (placebo or active), we used the term the number needed to harm or cause one additional harmful event (NNH).

The plan was to analyse data by type of clinical condition (postoperative dental pain, postoperative gynaecological pain, or dysmenorrhoea). Previous research suggested that the type of surgery made no difference in NNT estimates for acute postoperative pain [35], and this is largely supported by a more recent analysis [36].

Where there were sufficient data (defined as at least two trials and 200 patients [26]), we calculated RR and NNT with 95% confidence intervals. The risk ratio was calculated using a fixed-effect model [37] with no statistically significant difference between treatments assumed when the 95% confidence intervals included unity. NNT (or NNTp, NNH) was calculated [38] using the pooled number of observations only when there was a statistically significant difference of RR. The significance of any differences between pain condition, dose, and different analgesics was evaluated using the *z* test [1].

## 3. Results

### 3.1. Results of the Search

The Novartis database included eight randomised trials (Supplementary Appendix 1), three for pain following third molar extraction (519 patients, predominantly women; CSRs 02, 03, and 04), three after gynaecological surgery (679 women; CSRs 05, 06, and 07), and two in dysmenorrhoea (711 women; CSRs 10 and 11). All were conducted in the USA or Venezuela by the Ciba-Geigy pharmaceutical division from 1988 to 1990, with clinical trial reports all dated in 1991. Extensive enquiries failed to identify any trials with numbers 01, 08, or 09. No individual patient-level results were available. One published report with 151 patients [17] had a Ciba-Geigy author, but no clinical trial report could be found for that trial.

Searches for systematic reviews of diclofenac and diclofenac potassium for acute postoperative pain found a non-Cochrane review [39] that had been updated in four subsequent Cochrane reviews in 1999, 2004, 2009, and 2015 [13, 40–42]; the most recent had reported results for fast-acting soluble formulations, diclofenac potassium, and diclofenac sodium salts separately and included published versions of all four studies covered in the CSRs. It found nine diclofenac potassium studies with doses of 50 mg or 100 mg, though analysed some as fast-acting formulations (solution, softgel) [16, 17, 43–49]. Diclofenac for dysmenorrhoea was examined in a Cochrane systematic review originally published in 2010 and updated in 2015 [20, 50], but not an earlier review [51]. One large published report (also available as a CSR) reported only mean pain values from a graph [52]. The review presented no analysis of efficacy by diclofenac formulation or dose.

**Figure 1:**
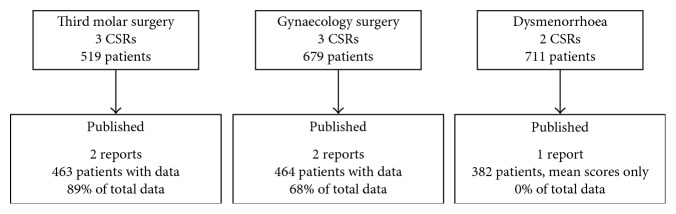
Breakdown of studies and patients identified in CSRs and published papers. CSR = clinical study reports.

**Figure 2:**
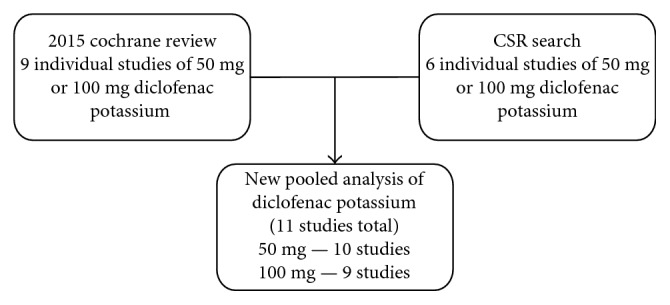
Flow diagram of search results for unique trials of diclofenac potassium 50 mg and 100 mg. CSR = clinical study reports.

We paired study methods and demographics of the CSRs with those of published trials, comparing numbers of patients per treatment group, mean age and range, sex distribution, and initial pain intensity (Supplementary Appendix 2). Three CSRs accounting for 599/1909 patients (31%) in the CSRs appear not to have been published (Figure 1). CSR 03 terminated early because of falsification problems in one centre (data not used in the CSR), and we could identify no published reports of CSRs 07 and 10. Moreover, it is worth noting that three identified published papers [18, 46, 47] (846/1909 patients (44%)) published in Today's Therapeutic Trends (a journal now defunct) do not appear in PubMed, the most likely place most people would search to find data. These had been identified in the Cochrane reviews because of information made available in a hand-searched database [21]; they can be found in the Cochrane Central Register of Controlled Trials (CENTRAL) because that incorporates the hand-searched database and in EMBASE.  Figure 2 shows the flow diagram for individual studies available for an updated pooled analysis of 50 mg and 100 mg diclofenac potassium in postoperative pain.

Of the 1909 patients enrolled in the original Ciba-Geigy trials in the late 1980s and early 1990s, data from 1445 (76%) were either unpublished or published in journals not covered by PubMed.

### 3.2. Risk of Bias Evaluations

Pairwise comparisons for the risk of bias evaluations are in Supplementary Appendix 3. Using the Oxford Quality Score, all CSRs scored the maximum of 5/5, compared with scoring 3/5 to 5/5 in the published versions. All CSRs had low risk of bias scores for random sequence generation (all used a computer-generated random list), allocation concealment (stratified, sequential allocation), and blinding (double dummy technique). The published papers had less detail, leading to an assessment of a higher, unclear, grade of risk of bias for these items (Supplementary Appendix 3). It was notable that the eight study reports comprised 1451 pages in all (average 181 per report), far more than the published reports, which varied between 8 and 17 pages (average 13 per report).

### 3.3. Data Extraction

Supplementary Appendix 4 has details extracted from all CSRs, including details of participants, drugs administered, study design, quality scores, and results for pain, pain relief, withdrawals, and adverse events. All CSRs were full reports; the report for CSR 03 excluded data from one centre discovered to have falsified data.

### 3.4. Analysis of Data Available in CSRs

#### 3.4.1. Analgesic Results in CSRs of Dental Studies

Information was available from 509 patients, with detailed results for at least 50% maximum pain relief, global evaluation of “very good or excellent” and patients remedicating within 8 hours (Table 1). All three trials used placebo, with aspirin 650 mg as a common active comparator. Comparisons of active treatments with placebo are shown in Table 1.

NNT values for diclofenac potassium 50 and 100 mg were low, at about 2, for the two efficacy results of at least 50% maximum pain relief and global evaluation of “very good or excellent,” with somewhat better results for the higher dose, although the difference was not significant. Fewer patients remedicated with active treatments than with placebo, with the best results obtained for diclofenac potassium 100 mg.

**Figure 3:**
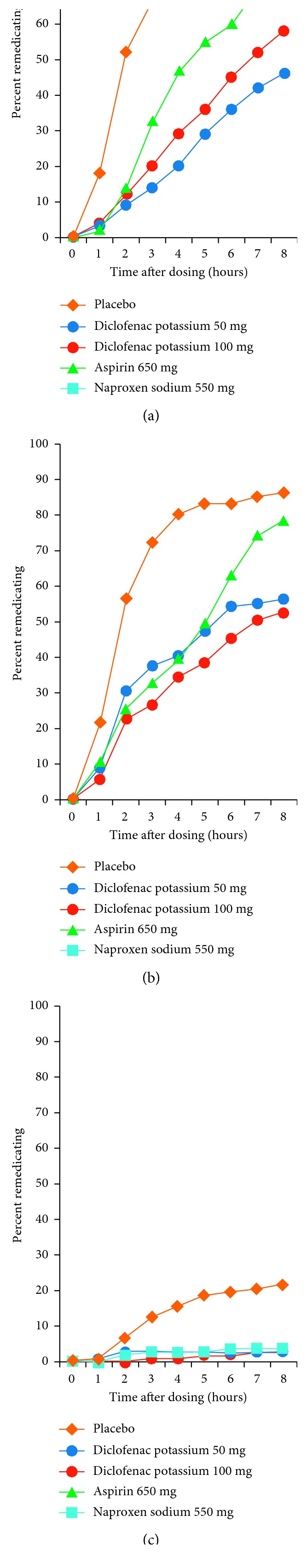
Remedication over time following third molar surgery (a) and gynaecological surgery (b) and in dysmenorrhoea (c) following administration of drugs to patients with moderate or severe pain.

Diclofenac potassium 100 mg was significantly better than aspirin 650 mg on three efficacy measures, but 50 mg was not (Table 1). The time for half of the patients to remedicate with diclofenac potassium 50 mg and 100 mg was longer, at about seven hours or more, than for aspirin 650 mg at about 4 hours, and placebo at 1-2 hours (Figure 3).

#### 3.4.2. Analgesic Results in CSRs of Gynaecological Studies

Information was available from 685 patients, with results for at least 50% maximum pain relief, global evaluation of “very good or excellent,” and patients remedicating within 8 hours (Table 2). Both trials used placebo, with aspirin 650 mg as a common active comparator. Comparisons of active treatments with placebo are shown in Table 2.

NNT values for diclofenac potassium 50 mg and 100 mg were low, at about 2.2 to 2.5, for the two efficacy results of at least 50% maximum pain relief and global evaluation of “very good or excellent,” with somewhat better results for the lower dose, although the difference was not significant. Fewer patients remedicated with active treatments than with placebo, with the best results for diclofenac potassium 100 mg.

Diclofenac potassium 50 mg was significantly better than aspirin 650 mg on the three efficacy measures, and 100 mg was significantly better than aspirin 650 mg for global evaluation and remedication (Table 2). The time for half of the patients to remedicate was similar to that of diclofenac potassium 50 mg and 100 mg and for aspirin 650 mg (at about 5–7 hours), but longer than placebo at 1-2 hours (Figure 3).

#### 3.4.3. Analgesic Results in CSRs of Dysmenorrhoea

Information was available from 711 women, but trials included data from two menstrual cycles in almost all of the women. Both trials used placebo and naproxen sodium 550 mg as a common active comparator.

Because there were no washout issues, there was a fresh randomisation for each cycle; as results of first and second cycle studies were not different, these have been combined in this analysis. Results for the first dose of analgesic per cycle have been used in this report. The comparisons of active treatments with placebo are shown in Table 3.

NNT values for diclofenac potassium 50 mg and 100 mg were modest, at about 3.0 to 4.0, for the two efficacy results of at least 50% maximum pain relief and global evaluation of “very good or excellent,” with somewhat better results for the higher dose, though the difference was not significant. Fewer patients remedicated with active treatments than with placebo, with best results for 100 mg.

Neither dose of diclofenac potassium was significantly better than naproxen sodium 550 mg on any efficacy measure (Table 3). Remedication rates were very low with all three active drugs and much lower than with placebo. In no group did half the patients remedicate (Figure 3).

#### 3.4.4. Adverse Events

None of the comparisons for adverse events found any difference between active drug and placebo for the number of patients experiencing an adverse event after a single dose of study medication (Tables 1 [Table tab2]–3). Event rates with placebo were below 10% for postoperative dental and gynaecological pain when collected over the period of a single dose. They were 37%–48% in dysmenorrhoea studies, reflecting adverse event collections over a much longer interval for the whole time between cycles.

No serious adverse events were reported in the CSR or published report of any postoperative trial. In the dysmenorrhoea trials, one patient in a naproxen sodium group had a diagnosis of breast cancer (CSR 10). CSR 11 reported several serious events. In diclofenac groups, these were one probable and one possible seizure, and one each of dulled sensorium, erosive gastritis, increased bilirubin, ureteral calculus, and appendicitis. In the naproxen group, there was one death in a fire and one case of Bell's palsy.

### 3.5. Analysis of All Available Postoperative Data

We updated the calculations of at least 50% maximum pain relief, patients remedicating within 8 hours, and those who experienced at least one adverse event for all available postoperative data. For the 50 mg and 100 mg dose, this analysis used data from seven trials with diclofenac potassium [17, 27, 31, 36, 43–45] together with additional data from diclofenac potassium in a soluble formulation [45, 49] and data from unpublished CSRs (CSRs 03 and 07). The results are shown in Table 4, together with comparable pooled analyses from all six CSRs in order to make comparisons with results of the Cochrane review [13]. Results were almost identical for each outcome and dose of these three analyses.

## 4. Discussion

This study examined CSRs of eight randomised trials (1909 patients), investigating the effects of diclofenac potassium in acute postoperative pain after dental or gynaecological surgery and dysmenorrhoea. Analysis of CSRs for postoperative pain agreed with analyses of extant systematic reviews, demonstrating that diclofenac potassium was an effective analgesic using outcomes of at least 50% of maximum pain relief, global evaluation of “very good or excellent,” and the need for remedication for inadequate pain relief within 8 hours. NNT values were low and comparable with analgesics known to be very effective in these conditions. There was no clinically important difference in efficacy between 50 mg and 100 mg doses of diclofenac potassium; NNTs for 50 mg were almost identical to those for 100 mg (Table 4).

Although the majority of data from these CSRs had been published in peer-reviewed journals, data from three had not; this included data from 547 patients in fully completed trials and 56 from a discontinued trial (32% of the total). Unfortunately, much of the published information was in an obscure journal, now discontinued, and not available in the most-used search engine of PubMed, but only through specialist registers or through EMBASE, which is less frequently used for casual searches because it often requires subscription. None of these trials would be found by casual, or perhaps even quite informed searching of electronic databases, meaning that around 76% of available evidence from these CSRs would not be easily available. The fact that the Cochrane reviews in acute pain [13] and dysmenorrhoea [20] had identified all the published studies reflected an extensive exercise in hand-searching literature and the value of the Cochrane Central Register of Controlled Trials (CENTRAL). One trial with a pharmaceutical company-associated author-reported data from 151 patients was available in a published form without an identified CSR. The reasons are unknown.

### 4.1. Quality of Reporting of Clinical Trials in CSRs and Published Reports

The quality of the evidence available in the CSRs was good. All of the trials were properly randomised and blinded and scored a maximum of 5/5 on the Oxford Quality Score. Risk of bias assessment typically conducted in the Cochrane reviews showed a general absence of risk, with the exception of small trial size; small acute pain trials like these are susceptible to random chance effects [26].

The longer, more detailed CSRs (average 181 pages per report compared with 13 published pages on average) contained more information relevant to assessment of study quality and risk of bias than did published versions, which omitted important methodological information. Assessment of published evidence was downgraded because of these omissions and judged to be at a potentially higher risk of bias than was actually the case. Compared with published versions of the trials, the CSRs provided additional important methodological insights and reassurance about lower risks of bias.

It is worth noting, however, that the trial funding source or statements of conflicts of interest potentially influence readers more than the actual quality of the evidence [53, 54]. While there may well be unease about industry-funded studies [55], for acute pain there is good evidence of no influence of industry funding on study results [56].

### 4.2. Estimates of Efficacy Compared with Published Reviews

For postoperative pain, an updated Cochrane review of diclofenac for acute postoperative pain is the most relevant published analysis [13], and the updated 2016 analysis is the most recent comprehensive analysis. Table 4 compares the CSR results with both of these. For the outcomes of at least 50% of maximum pain relief, the proportion of patients remedicating, and patients experiencing at last one adverse event, NNTs for each analysis at both 50 mg and 100 mg were very close to one another, with at most a difference of 0.3 in point estimates. Results first available from trials completed in 1990 were essentially the same as the totality of trial data available in 2016.

Corresponding results for CSR analyses and larger systematic reviews were also seen for aspirin. For the six postoperative CSRs in this review, the NNT for at least 50% maxTOTPAR for aspirin 650 mg compared with placebo, with 543 patients in the comparison, was 3.2 (2.6 to 4.1). This is not statistically different from the NNT from a Cochrane review for aspirin 600/650 mg reporting 4.2 (3.9 to 4.8) based on 4630 patients from 60 studies in the comparison with placebo [57]. The NNTp for remedication over eight hours for aspirin 650 mg was 7.1 (4.6 to 15) in the six CSRs, similar to the NNTp of 5.1 (4.2 to 6.5) based on 1838 patients in 20 studies for six- to eight-hour remedication rates.

Neither the Cochrane review nor this review found any difference between active drug and placebo in terms of patients reporting adverse events after a single dose. For both diclofenac potassium at 50 mg and 100 mg doses and the comparator, aspirin 650 mg, the results from clinical study reports provide essentially the same results for efficacy and for adverse event reporting.

A Cochrane review on NSAIDs for dysmenorrhoea was able to analyse data from three small studies of 140 women taking diclofenac for dysmenorrhoea, with no mention of the formulation used (one was probably a suppository), and reporting only odds ratios [20]. This review of CSRs was able to perform a series of analyses on efficacy and harm (Table 3) based on information from 711 women, most of whom reported results for two menstrual cycles. In terms of dysmenorrhoea, therefore, the current review is the first to provide any useful information for diclofenac potassium.

Naproxen sodium 550 mg was reported to have an NNT of 3.1 (2.4 to 4.4) for the outcome of at least 50% pain relief over six hours in a pooled analysis of two trials of 359 menstrual cycles in 231 women [11]. This compares with an NNT of 3.5 (2.9 to 4.6) for naproxen sodium 550 mg for the same outcome from 784 cycles in this review of CSRs of different trials. We could not make any comparison with naproxen between this review and the Cochrane review [20], which used an outcome of at least moderate pain relief, a lesser outcome than here; it also reported only a comparison of odds ratios. The Cochrane review reported a pooled odds ratio of 3.7 (2.9 to 4.6) for 16 studies. We calculated for dysmenorrhoea an odds ratio of 3.1 (2.4 to 4.2) for at least 50% maximum pain relief for the CSRs, suggesting a similar effect size for naproxen in the two analyses.

## 5. Conclusions

For postoperative pain and especially for dysmenorrhoea, review of clinical study reports of diclofenac potassium has produced substantial additional information to that available in current Cochrane reviews, the only published systematic reviews we found relevant to diclofenac potassium in these painful conditions. Clinical study reports provided more detailed descriptions of methods used in trials and showed that the trials were of higher quality and with lower risk of bias than those reported in published accounts of the same trials. These findings indicate that the clinical study reports provide a better appreciation of efficacy and harm than the published reports in acute painful conditions. This has been shown before for acute [5, 8–11, 14, 28] and chronic pain conditions [3, 4, 6].

Where quantitative comparison was possible for postoperative conditions, the main efficacy results from review of these CSRs provided very similar estimates to the published systematic reviews, with no tendency to under- or overestimation. Once a minimum amount of good quality clinical trial data are available, then there is little tendency for the effect size to change when similar trials are performed. This has implications for the need to continually update some systematic reviews, at least for single dose studies in acute pain that have standard and validated methods [58]. It also means we can put even greater trust in the totality of available evidence in acute pain [59].

Where there are limited data on particular formulations for analgesics used to treat acute pain, as for diclofenac potassium, the use of CSRs provided more data and allowed calculation of outcomes of known value [29]. The value of those outcomes may not have been appreciated when the studies were designed and conducted, over 25 years ago. The result was to generate greater confidence in the value of the diclofenac potassium formulation to treat acute painful conditions. For dysmenorrhoea particularly, only information from CSRs provided a meaningful insight into drug effects in that condition.

## Figures and Tables

**Table 1:** Efficacy and adverse event data for postoperative dental studies.

Outcomes	Percent with outcome		Risk ratio (95% CI)	NNT, NNTp, or NNH (95% CI)	Comparison with aspirin 650 mg
Drug and dose	Active	Placebo			
*At least 50% maximum pain relief (TOTPAR)*					
Diclofenac-K 50 mg	57	9	6.4 (5.1 to 15)	2.1 (1.7 to 2.6)	*p*=0.072
Diclofenac-K 100 mg	68	9	7.7 (4.3 to 14)	1.7 (1.4 to 2.0)	^∗^ *p=*0.008
Aspirin 650 mg	44	9	5.0 (2.7 to 9.1)	2.9 (2.2 to 4.1)	
*Global evaluation—“very good or excellent”*					
Diclofenac-K 50 mg	54	8	6.7 (3.5 to 13)	2.2 (1.8 to 2.8)	*p*=0.052
Diclofenac-K 100 mg	65	8	8.2 (4.4 to 15)	1.8 (1.5 to 2.1)	^∗^ *p=*0.004
Aspirin 650 mg	40	8	5.0 (2.6 to 9.5)	3.2 (2.4 to 4.7)	
*Patients who remedicate within 8 hours*				*NNTp*	
Diclofenac-K 50 mg	59	85	0.7 (0.6 to 0.8)	3.7 (2.6 to 6.3)	*p*=0.121
Diclofenac-K 100 mg	45	85	0.5 (0.4 to 07)	2.5 (2.0 to 3.4)	^∗^ *p=*0.0011
Aspirin 650 mg	71	85	0.8 (0.7 to 0.9)	6.8 (4.0 to 23)	
*Patients experiencing any adverse event*				*NNH*	
Diclofenac-K 50 mg	10	9	1.1 (0.5 to 2.5)	Not calculated	
Diclofenac-K 100 mg	13	9	1.4 (0.7 to 3.0)	Not calculated	
Aspirin 650 mg	10	9	1.0 (0.5 to 2.2)	Not calculated	

Note: comparisons were carried out using a two-tailed *z*-test (Tramer et al. [1]). ^∗^statistical significance; NNT = number needed to treat for one to benefit; NNTp = number needed to treat to prevent one event; NNH = number needed to treat for one to be harmed.

**Table 2 tab2:** Efficacy and adverse event data for postoperative gynaecology studies.

Outcomes	Percent with outcome		Risk ratio (95% CI)	NNT, NNTp, or NNH (95% CI)	Comparison with aspirin 650 mg
Drug and dose	Active	Placebo			
*At least 50% maximum pain relief (TOTPAR)*					
Diclofenac-K 50 mg	60	17	3.6 (2.5 to 5.2)	2.3 (1.9 to 3.0)	^∗^ *p=*0.044
Diclofenac-K 100 mg	56	17	3.4 (2.3 to 4.9)	2.5 (2.0 to 3.4)	*p*=0.13
Aspirin 650 mg	46	17	2.8 (1.9 to 4.1)	3.5 (2.6 to 5.2)	
*Global evaluation—“very good or excellent”*					
Diclofenac-K 50 mg	59	13	4.4 (2.9 to 6.7)	2.2 (1.8 to 2.8)	^∗^ *p=*0.0023
Diclofenac-K 100 mg	54	13	4.0 (2.7 to 6.1)	2.5 (2.0 to 3.2)	^∗^ *p=*0.021
Aspirin 650 mg	38	13	2.9 (1.8 to 4.5)	4.0 (2.9 to 6.4)	
*Patients who remedicate within 8 hours*				*NNTp*	
Diclofenac-K 50 mg	37	71	0.5 (0.4 to 06)	2.9 (2.2 to 4.2)	^∗^ *p=*0.0061
Diclofenac-K 100 mg	39	71	0.5 (0.4 to 0.7)	3.1 (2.3 to 4.5)	^∗^ *p=*0.012
Aspirin 650 mg	58	71	08 (0.7 to 0.9)	7.4 (4.2 to 34)	
*Patients experiencing any adverse event*				*NNH*	
Diclofenac-K 50 mg	8	5	1.5 (0.6 to 3.4)	Not calculated	
Diclofenac-K 100 mg	6	5	1.2 (0.5 to 2.9)	Not calculated	
Aspirin 650 mg	9	5	1.9 (0.8 to 4.3)	Not calculated	

Note: comparisons were carried out using a two-tailed *z*-test (Tramer et al. [1]). ^∗^statistical significance; NNT = number needed to treat for one to benefit; NNTp = number needed to treat to prevent one event; NNH = number needed to treat for one to be harmed.

**Table 3:** Efficacy and adverse event data for dysmenorrhoea studies.

Outcomes	Percent with outcome		Risk ratio (95% CI)	NNT, NNTp, or NNH (95% CI)	Comparison with naproxen 550 mg
Drug and dose	Active	Placebo			
*At least 50% maximum pain relief (TOTPAR)*					
Diclofenac-K 50 mg	54	28	1.9 (1.5 to 2.6)	3.8 (2.8 to 6.1)	*p*=0.73
Diclofenac-K 100 mg	64	30	2.1 (1.8 to 2.5)	3.0 (2.5 to 3.7)	*p*=0.30
Naproxen 550 mg	59	30	1.9 (1.6 to 2.3)	3.5 (2.9 to 4.6)	
*Global evaluation—“very good or excellent”*					
Diclofenac-K 50 mg	49	20	2.5 (1.8 to 3.4)	3.5 (2.6 to 5.1)	*p*=0.21
Diclofenac-K 100 mg	47	18	2.6 (2.0 to 3.3)	3.5 (2.9 to 4.5)	*p*=0.13
Naproxen 550 mg	40	18	2.2 (1.7 to 2.8)	4.6 (3.6 to 6.4)	
*Patients who remedicate within 8 hours*				*NNTp*	
Diclofenac-K 50 mg	3	17	0.2 (0.1 to 0.4)	7.0 (4.9 to 12)	*p*=0.64
Diclofenac-K 100 mg	3	20	0.1 (0.08 to 0.3)	5.6 (4.5 to 7.5)	*p*=0.66
Naproxen 550 mg	4	20	0.2 (0.1 to 0.4)	6.2 (4.8 to 8.5)	
*Patients experiencing any adverse event*				*NNH*	
Diclofenac-K 50 mg	49	48	1.0 (0.8 to 1.4)	Not calculated	
Diclofenac-K 100 mg	38	37	1.0 (0.8 to 1.3)	Not calculated	
Naproxen 550 mg	40	37	1.1 (0.8 to 1.4)	Not calculated	

Note: comparisons were carried out using a two-tailed *z*-test [50]. NNT = number needed to treat for one to benefit; NNTp = number needed to treat to prevent one event; NNH = number needed to treat for one to be harmed.

**Table 4:** Comparison between results of Cochrane reviews and CSR pooled data.

Dose (mg)	Number of studies	Number of Participants	Percent with outcome		Risk ratio (95% CI)	NNT or NNTp (95% CI)
			Diclofenac	Placebo		
*At least 50% maximum pain relief 50* *mg diclofenac potassium*						*NNT*
Max trials 2016	10	1083	60	14	3.9 (3.1 to 4.9)	2.2 (2.0 to 2.5)
Cochrane review	7	757	64	17	3.7 (2.9 to 4.7)	2.1 (1.9 to 2.5)
CSR data	6	542	59	13	4.4 (3.2 to 6.1)	2.2 (1.9 to 2.6)
*100* *mg diclofenac potassium*						
Max trials 2016	9	847	59	12	5.0 (3.8 to 6.6)	2.1 (1.9 to 2.4)
Cochrane review	5	589	65	13	4.8 (3.6 to 6.5)	1.9 (1.7 to 2.2)
CSR data	6	544	62	13	4.6 (3.4 to 6.3)	2.1 (1.8 to 2.4)
*Patients who remedicate within 8 hours 50* *mg diclofenac potassium*						*NNTp*
Max trials 2016	10	1083	41	75	0.55 (0.49 to 0.61)	2.9 (2.5 to 3.5)
Cochrane review	7	757	36	69	0.52 (0.45 to 0.60)	3.0 (2.5 to 3.8)
CSR data	6	542	46	77	0.60 (0.53 to 0.68)	3.2 (2.6 to 4.3)
*100* *mg diclofenac potassium*						
Max trials 2016	9	847	42	78	0.54 (0.48 to 0.61)	2.8 (2.4 to 3.4)
Cochrane review	5	589	34	72	0.45 (0.38 to 0.54)	2.6 (2.2 to 3.3)
CSR data	6	544	42	77	0.54 (0.47 to 0.62)	2.8 (2.3 to 3.6)
*Patients with at least one adverse event 50* *mg diclofenac potassium*						*NNH*
Max trials 2016	10	1095	6.9	8.2	0.94 (0.62 to 1.4)	Not calculated
Cochrane review	7	778	6.8	7.6	0.94 (0.55 to 1.6)	Not calculated
CSR data	6	555	8.9	7	1.3 (0.73 to 2.3)	Not calculated
*100* *mg diclofenac potassium*						
Max trials 2016	9	861	9.6	8.5	1.1 (0.72 to 1.7)	Not calculated
Cochrane review	5	509	8.5	9.1	0.89 (0.46 to 1.7)	Not calculated
CSR data	6	557	9	6.8	1.3 (0.74 to 2.3)	Not calculated

CSR = clinical study report; NNT = number needed to treat for one to benefit; NNTp = number needed to treat to prevent one event; NNH = number needed to treat for one to be harmed. Cochrane review is that of Derry 2015 [17].

## Data Access

The data from studies used for the analysis are provided in the manuscript and the supporting files.

## Supplementary Materials

Supplementary Materials These consist of four appendices containing datasets that are related to the manuscript. *Appendix 1.* Clinical study reports and any published version. *Appendix 2.* Information regarding data used to match clinical study reports and publications. *Appendix 3.* Detailed quality scores and risk of bias for clinical study reports and publications. *Appendix 4.* Digest of clinical trials methods, with efficacy and adverse event results.
