# Supplementary material for: Diclofenac Potassium in Acute Postoperative Pain and Dysmenorrhoea: Results from Comprehensive Clinical Trial Reports
Source: Pain Res Manag. 2018 Jan 17;2018:9493413. doi: 10.1155/2018/9493413 (PMC5829436; doi:10.1155/2018/9493413)
Supplement: Supplementary Materials — These consist of four appendices containing datasets that are related to the manuscript. Appendix 1. Clinical study reports and any published version. Appendix 2. Information regarding data used to match clinical study reports and publications. Appendix 3. Detailed quality scores and risk of bias for clinical study reports and publications. Appendix 4. Digest of clinical trials methods, with efficacy and adverse event results. [file 9493413.f1.pdf]

Appendix 1: Clinical study reports and linked publications

Clinical study reports (CSRs)	Title and lead investigator	Linked publications
**CSR 02**	A double-blind study comparing the safety and efficacy of diclofenac potassium, aspirin, or placebo in the treatment of moderate or severe pain secondary to dental impaction surgery. Report date January 15 1991. Lead investigator S Nelson, Columbia, MD, USA.	Nelson S, Brahim J. An evaluation of the analgesic efficacy of diclofenac potassium, aspirin, and placebo in postoperative dental pain. Today's Therapeutic Trends 1994;12:3–14.
**CSR 03**	A double-blind study comparing the safety and efficacy of diclofenac potassium, aspirin, or placebo in the treatment of moderate or severe pain secondary to dental impaction surgery. Report date February 11 1991. Lead investigator SA Cooper, Bryn Mawr, PA, USA.	No published report identified.
**CSR 04**	A double-blind study comparing the safety and efficacy of diclofenac potassium, aspirin, or placebo in the treatment of moderate or severe pain secondary to dental impaction surgery. Report date February 11 1991. Lead investigator DR Mehlisch, Austin, Tx, USA.	Mehlisch DR, Brown P. Single-dose therapy with diclofenac potassium, aspirin or placebo following dental impaction surgery. Today's Therapeutic Trends 1995;12(Suppl 1): 15–31.
**CSR 05**	The comparative efficacy of diclofenac potassium, aspirin, or placebo in the treatment of post-episiotomy pain. Report date March 7 1990.	Olson NZ, Sunshine A, Zighelboim I, DeCastro A. Onset and duration of analgesia of diclofenac

	Lead investigator A Sunshine, I Zighelboim. New Yrk, NY and Caracas, Venezuela.	potassium in the treatment of postepisiotomy pain. American Journal of Therapeutics 1997;4:239–46.
**CSR 06**	The comparative efficacy of diclofenac potassium, aspirin, and placebo in the treatment of pain secondary to gynaecological surgery. Report date June 4 1990.<br>Lead investigator R Herbetson, Salt Lake City, Utah, USA.	Herbertson RM, Storey N. The comparative efficacy of diclofenac potassium, aspirin and placebo in the treatment of patients with pain following gynecologic surgery. Today's Therapeutic Trends 1995;12(Suppl 1):33–45.
**CSR 07**	The comparative efficacy of diclofenac potassium, aspirin, and placebo in the treatment of post-gynaecological surgery pain. Report date February 11 1991.<br>Lead investigator G Wideman, Birmingham, Alabama, USA.	No published report identified.
**CSR 10**	A double-blind, comparative study of diclofenac potassium vs. naproxen sodium vs placebo in the treatment of primary dysmenorrhea. Report date May 6 1991.<br>Lead investigator J Benz – multicentre study with US investigators)	No published report identified.
**CSR 11**	A double-blind, comparative study of diclofenac potassium (with and without a loading dose) vs. naproxen sodium vs placebo in the treatment of primary dysmenorrhea. Report date May 6 1991.<br>Lead investigator R Anderson – multicentre study with US investigators).	Kintigh JW. A multicenter, randomized, double-blind, placebo controlled study of diclofenac potassium versus naproxen sodium in the treatment of primary dysmenorrhea. Today's

	Therapeutic Trends 1995;12 Supplement(1):47–61.

CSR = clinical study report

Appendix 2: CSRs and matching with published reports

Individual studies	Dates of trial and report	Number of patients	Age (years ± SD)	Sex distribution	Initial pain intensity
**Dental studies**					
CSR 02	1988<br>Report 1991	255	25.4 ± 6.6<br>Range 16-57	51% F<br>49% M	90% moderate<br>10% severe
Nelson 1994<br>[49]	1994	255	25	51% F<br>49% M	90% moderate<br>10% severe
CSR 03	1988<br>Report 1990 and 1991	56	22.8 ± 4.7<br>Range 16-35	62% F<br>38% M	57% moderate<br>43% severe
No identified publication					
CSR 04	1988<br>Report 1989 and 1991	208	25.7 ± 6.9<br>Range 16-70	56% F<br>44% M	72% moderate<br>28% severe
Mehlish 1995<br>[33]	1995	208	25.7<br>Range 16-70	56% F<br>44% M	72% moderate<br>28% severe
**Gynaecological studies**					
CSR 05	1989<br>Report 1990	255	23.9 ± 5.2<br>Range 18-46	100% F	0% moderate<br>100% severe
Olson 1997<br>[51]	1997	255	23.9	100% F	0% moderate<br>100% severe
CSR 06	1989	209	43.4 ± 13.2	100% F	83% moderate

	Report 1990		Range 16-78		17% severe
Herbertson 1995 [19]	1995	209	43.4	100% F	83% moderate<br>17% severe
CSR 07	1988<br>Report 1989 and 1991	215	34.7 ± 9.0<br>Range 18-66	100% F	86% moderate<br>14% severe
No identified publication					
**Dysmenorrhoea studies**					
CSR 10	1989-90<br>Report 1991	328	29.7 ± 6.2<br>Range 16-43	100% F	61% moderate<br>39% severe
No identified publication					
CSR 11	1989-90<br>Report 1991	383	30.3 ± 6.1<br>Range 16-42	100% F	70% moderate<br>30% severe
Kintigh 1995 [28]	1995	382	16-42	100% F	

CSR = clinical study report

Appendix 3: Quality score and risk of bias for CSRs and matched publications

Studies	Oxford Quality Score	Random sequence generation (selection bias)	Allocation concealment (selection bias)	Blinding of participants and personnel (performance bias)	Size	Pages
**Dental studies**						
CSR 02	R2, DB2, W1	Low	Low	Low	High	110
Nelson 1994 [49]	R1, DB2, W1	Unclear	Unclear	Low	High	12
CSR 03	R2, DB2, W1	Low	Low	Low	High	251
No identified publication						
CSR 04	R2, DB2, W1	Low	Low	Low	High	204
Mehlish 1995 [33]	R1, DB2, W1	Unclear	Unclear	Low	High	17
**Gynaecological studies**						
CSR 05	R2, DB2, W1	Low	Low	Low	High	74
Olson 1997 [51]	R1, DB2, W1	Unclear	Unclear	Low	High	8
CSR 06	R2, DB2, W1	Low	Low	Low	Unclear	84
Herbertson 1995 [19]	R1, DB1, W1	Unclear	Unclear	Unclear	Unclear	13

CSR 07	R2, DB2, W1	Low	Low	Low	High	87
No identified publication						
**Dysmenorrhoea studies**						
CSR 10	R2, DB2, W1	Low	Low	Low	Unclear	263
No identified publication						
CSR 11	R2, DB2, W1	Low	Low	Low	Unclear	378
Kintigh 1995 [28]	R1, DB2, W1	Unclear	Unclear	Low	Unclear	15

The Oxford Quality score is based on randomisation (0, 1, or 2 points), double blinding (0, 1, or 2 points), and withdrawals (0 or 1 point). CSR = clinical study report; R = randomisation; DB = double blinding; W = withdrawals.

Appendix 4: Details of clinical trial methods, and results for efficacy and adverse events from CSRs

Clinical study report	Participants and condition	Drug	Design	Oxford QS	Pain relief	Patients with ≥50%max TOTPAR	Patient Global	Median time to remed (h)	Patients remedicating	Assessment of AE	Any AE	Withdrawal	Serious AE
**Dental pain studies**													
**CSR 02<br>Study 1988<br>Report 1991<br><br>Nelson 1994<br>[49]**	Moderate to severe pain within 12 hours of third molar extraction<br>Age 16-70 years<br>Mean age 25 years<br>M or F (51% F)	Diclofenac-K 25 mg, n = 52<br>Diclofenac-K 50 mg, n = 51<br>Diclofenac-K 100 mg, n = 50<br>Aspirin 650 mg, n = 50<br>Placebo, n = 52	Randomised using computer generated sequence of random numbers<br>Double blind, double dummy, with identical appearance of treatments<br>Blind maintained until after trial concluded<br>Pain assessed 0.5, 1, 2, 3, 4, 5, 6, 7, 8 hours<br>Outcomes: standard 4 point PI scale, 5 point PR scale and 5 point patient global assessment scale	R = 2<br>DB = 2<br>W = 1<br>Total = 5/5<br><br>RoB size unclear	TOTPAR 6:<br>DK 25 = 10.8<br>DK 50 = 12.5<br>DK 100 = 15.2<br>ASA = 11.6<br>Pbo = 4.0	DK 25 = 25/51<br>DK 50 = 30/51<br>DK 100 = 36/50<br>ASA = 26/49<br>Pbo = 6/52	Very good or excellent<br>DK 25 = 24/51<br>DK 50 = 26/51<br>DK 100 = 34/50<br>ASA = 26/49<br>Pbo = 7/51	DK 25 = 5.5<br>DK 50 = >8<br>DK 100 = >8<br>ASA = 8<br>Pbo = 2.5	By 8 hours<br>DK 25 = 32/52<br>DK 50 = 24/51<br>DK 100 = 19/50<br>ASA = 25/50<br>Pbo = 39/52	Not given	DK 25 = 5/52<br>DK 50 = 4/51<br>DK 100 = 4/50<br>ASA = 4/50<br>Pbo = 6/52	3 of originally randomised not included - 2 with no data and 1 lost to follow up	None reported
**CSR 03<br>Study 1988<br>Report 1991<br>Note that study discontinued due to data falsification at one site (data not included in analyses)**	Moderate to severe pain within 12 hours of third molar extraction<br>Age 16-70 years<br>Mean age 23 years<br>M or F (62% F)	Diclofenac-K 50 mg, n = 12<br>Diclofenac-K 100 mg, n = 16<br>Aspirin 650 mg, n = 15<br>Placebo, n = 13	Randomised using computer generated sequence of random numbers<br>Double blind, double dummy, with identical appearance of treatments<br>Blind maintained until after trial concluded<br>Pain assessed 0.5, 1, 2, 3, 4, 5, 6, 7, 8 hours<br>Outcomes: standard 4 point PI scale, 5 point PR scale and 5 point patient global assessment scale	R = 2<br>DB = 2<br>W = 1<br>Total = 5/5<br><br>RoB size high	TOTPAR 6:<br>DK 50 = 10.7<br>DK 100 = 11.1<br>ASA = 5.8<br>Pbo = 2.7	DK 50 = 6/12<br>DK100 = 8/16<br>ASA = 3/15<br>Pbo = 0/13	Very good or excellent<br>DK 50 = 7/12<br>DK 100 = 6/16<br>ASA = 2/15<br>Pbo = 0/13	DK 50 = 6<br>DK 100 = 5<br>ASA = 2.8<br>Pbo = 1.6	By 8 hours<br>DK 50 = 8/12<br>DK100 = 9/16<br>ASA = 13/15<br>Pbo = 12/13	Not given	DK 50 = 3/12<br>DK100 = 3/16<br>ASA = 0/15<br>Pbo = 1/15	3 patients lost to follow up	None reported
**CSR 04<br>Study 1988<br>Report 1991<br>Mehlisch 1995<br>[33]**	Moderate to severe pain within 12 hours of third molar extraction<br>Age 16-70 years<br>Mean age 26 years<br>M or F (56% F)	Diclofenac-K 50 mg, n = 53<br>Diclofenac-K 100 mg, n = 52<br>Aspirin 650 mg, n = 51<br>Placebo, n = 52	Randomised using computer generated sequence of random numbers<br>Double blind, double dummy, with identical appearance of treatments<br>Blind maintained until after trial concluded<br>Pain assessed 0.5, 1, 2, 3, 4, 5, 6, 7, 8 hours<br>Outcomes: standard 4 point PI scale, 5 point PR scale and 5 point patient global assessment scale	R = 2<br>DB = 2<br>W = 1<br>Total = 5/5<br><br>RoB size unclear	TOTPAR 6:<br>DK 50 = 12.2<br>DK 100 = 14.9<br>ASA = 9.4<br>Pbo = 3.6	DK 50 = 30/53<br>DK100 = 37/52<br>ASA = 21/51<br>Pbo = 4/52	Very good or excellent<br>DK 50 = 29/53<br>DK 100 = 37/52<br>ASA = 17/51<br>Pbo = 2/52	DK 50 = 6<br>DK 100 = 8<br>ASA = 3.5<br>Pbo = 1.5	By 8 hours<br>DK 50 = 35/53<br>DK100 = 26/52<br>ASA = 44/51<br>Pbo = 49/52	Not given	DK 50 = 5/53<br>DK 100 = 8/52<br>ASA = 7/51<br>Pbo = 4/52	None	None reported
**Post gynaecological surgery pain studies**													
**CSR 05<br>Study 1988<br>Report 1990<br><br>Olson 1997<br>[51]**	Moderate to severe pain within 49 hours of uncomplicated vaginal delivery with episiotomy<br>Age ≥18 years<br>Mean age 24 years	Diclofenac-K 25 mg, n = 52<br>Diclofenac-K 50 mg, n = 50<br>Diclofenac-K 100 mg, n = 51<br>Aspirin 650 mg, n = 50<br>Placebo, n = 52	Randomised using computer generated sequence of random numbers<br>Double blind, double dummy, with identical appearance of treatments<br>Blind maintained until after trial concluded<br>Pain assessed 0.5, 1, 2, 3, 4, 5, 6, 7, 8 hours<br>Outcomes: standard 4 point PI scale, 5 point PR scale and 5 point patient global assessment scale	R = 2<br>DB = 2<br>W = 1<br>Total = 5/5<br><br>RoB size unclear	TOTPAR 6:<br>DK 25 = 13.4<br>DK 50 = 14.8<br>DK 100 = 15.5<br>ASA = 11.5<br>Pbo = 7.5	DK 25 = 33/52<br>DK 50 = 35/50<br>DK100 = 38/51<br>ASA = 26/50<br>Pbo = 16/52	Very good or excellent<br>DK 25 = 24/52<br>DK 50 = 34/50<br>DK 100 = 39/51<br>ASA = 20/50<br>Pbo = 11/52	DK 25 = >8<br>DK 50 = >8<br>DK100 = >8<br>ASA = >8<br>Pbo = >8	By 8 hours<br>DK 25 = 2/52<br>DK 50 = 1/50<br>DK100 = 0/51<br>ASA = 5/50<br>Pbo = 19/52	Not given	DK 25 = 1/52<br>DK 50 = 0/50<br>DK100 = 1/51<br>ASA = 1/50<br>Pbo = 1/52	No withdrawals	None reported

**CSR 06 Study 1988 Report 1990 Herbertson 1995 [19]**	Moderate to severe pain within 96 hours of gynaecological surgery<br>Age ≥16 years<br>Mean age 43 years	Diclofenac-K 50 mg, n = 53<br>Diclofenac-K 100 mg, n = 53<br>Aspirin 650 mg, n = 54<br>Placebo, n = 54	Randomised using computer generated sequence of random numbers<br>Double blind, double dummy, with identical appearance of treatments<br>Blind maintained until after trial concluded<br>Pain assessed 0.5, 1, 2, 3, 4, 5, 6, 7, 8 hours<br>Outcomes: standard 4 point PI scale, 5 point PR scale and 5 point patient global assessment scale	R = 2<br>DB = 2<br>W = 1<br>Total = 5/5<br><br>RoB size unclear	TOTPAR 6:<br>DK 50 = 12.3<br>DK 100 = 12.2<br>ASA = 11.7<br>Pbo = 4.1	DK 50 = 29/52<br>DK 100 = 29/52<br>ASA = 28/53<br>Pbo = 6/52	Very good or excellent<br>DK 50 = 28/52<br>DK 100 = 25/52<br>ASA = 23/53<br>Pbo = 6/52	DK 50 = 6<br>DK100 = 5.5<br>ASA = 4.5<br>Pbo = 4	By 8 hours<br>DK 50 = 33/52<br>DK100 = 31/52<br>ASA = 46/53<br>Pbo = 44/52	Not given	DK 50 = 6/54<br>DK 100 = 6/55<br>ASA = 5/54<br>Pbo = 2/54	No withdrawals<br>A number of patients were not included in the pain analyses, principally because of rescue required before 1 hour, or vomiting after medicines taken<br>Episiotomy patients not included in efficacy analysis	None reported
**CSR 07 Study 1988 Report 1990**	Moderate to severe pain within 96 hours of gynaecological surgery<br>Age ≥16 years<br>Mean age 35 years	Diclofenac-K 50 mg, n = 55<br>Diclofenac-K 100 mg, n = 51<br>Aspirin 650 mg, n = 54<br>Placebo, n = 55	Randomised using computer generated sequence of random numbers<br>Double blind, double dummy, with identical appearance of treatments<br>Blind maintained until after trial concluded<br>Pain assessed 0.5, 1, 2, 3, 4, 5, 6, 7, 8 hours<br>Outcomes: standard 4 point PI scale, 5 point PR scale and 5 point patient global assessment scale	R = 2<br>DB = 2<br>W = 1<br>Total = 5/5<br><br>RoB size unclear	TOTPAR 6:<br>DK 50 = 11.8<br>DK100 = 10.3<br>ASA = 11.0<br>Pbo = 4.0	DK 50 = 28/52<br>DK100 = 23/50<br>ASA = 26/53<br>Pbo = 5/52	Very good or excellent<br>DK 50 = 29/52<br>DK100 = 19/50<br>ASA = 17/53<br>Pbo = 4/52	DK 50 = >8<br>DK100 = 4<br>ASA = 5.5<br>Pbo = 3.9	By 8 hours<br>DK 50 = 23/52<br>DK100 = 28/50<br>ASA = 39/53<br>Pbo = 48/52	Not given	DK 50 = 6/55<br>DK100 = 2/51<br>ASA = 9/54<br>Pbo = 5/55	No withdrawals<br>A number of patients were not included in the pain analyses, principally because of rescue required before 1 hour, or vomiting after medicines taken<br>Episiotomy patients not included in efficacy analysis	None reported
**Dysmenorrhoea studies**													
**CSR 10 Study 1989 Report 1991**	Moderate to severe pain associated with menstruation<br>Age 16-40 years<br>Mean age 30 years	First doses were:<br>Diclofenac-K 100 mg, n = 108<br>Naproxen 550 mg, n = 108<br>Placebo, n = 112<br>Initial dose of 2 tablets (DK 100 mg, Nap 550 mg, placebo), then 1 tablet every 6-8 hours, not exceeding 3 doses in any 24 hour period<br>Three day supply, for two cycles<br>Note: only first dose data	Randomised using computer generated sequence of random numbers<br>Double blind, double dummy, with identical appearance of treatments<br>Blind maintained until after trial concluded<br>Pain assessed 0.5, 1, 2, 3, 4, 5, 6, 7, 8 hours<br>Outcomes: standard 4 point PI scale, 5 point PR scale and 5 point patient global assessment scale, plus dysmenorrhoea symptom scorers	R = 2<br>DB = 2<br>W = 1<br>Total = 5/5<br><br>RoB size unclear	TOTPAR 6:<br>Cycle 1<br>DK 100 = 12.9<br>Nap 550 = 12.9<br>Pbo = 7.8<br>Cycle 2<br>DK 100 = 13.6<br>Nap 550 = 12.7<br>Pbo = 7.4	Cycle 1<br>DK 100 = 65/109<br>Nap 550 = 65/109<br>Pbo = 36/114<br>Cycle 2<br>DK 100 = 64/100<br>Nap 550 = 59/100<br>Pbo = 31/107	Very good or excellent<br>Cycle 1<br>DK 100 = 44/108<br>Nap 550 = 52/108<br>Pbo = 17/113<br>Cycle 2<br>DK 100 = 51/100<br>Nap 550 = 43/100<br>Pbo = 19/105	Cycle 1<br>DK100 = >8<br>Nap 550 = >8<br>Pbo = >8<br>Cycle 2<br>DK100 = >8<br>Nap 550 = >8<br>Pbo = >8	By 8 hours<br>Cycle 1<br>DK100 = 5/109<br>Nap 550 = 4/109<br>Pbo = 23/114<br>Cycle 2<br>DK 100 = 5/100<br>Nap 550 = 7/100<br>Pbo = 28/107	Not given	DK100 = 38/109<br>Nap 550 = 37/109<br>Pbo = 32/114	No withdrawals within first dose	No serious events evident in whole dosing schedule

**CSR 11<br>Study 1989<br>Report 1990**	Moderate to severe pain associated with menstruation<br>Age 16-40 years<br>Mean age 30 years	First doses were:<br>Diclofenac-K 100 mg, n = 100<br>Diclofenac-K 500 mg, n = 101<br>Naproxen 275 mg, n = 92<br>Placebo, n = 90<br><br>Initial dose of 2 tablets (DK 100 mg, Nap 550 mg, placebo), then 1 tablet every 6-8 hours, not exceeding 3 doses in any 24 hour period<br><br>Three day supply, for two cycles<br><br>Note: only first dose data	Randomised using computer generated sequence of random numbers<br>Double blind, double dummy, with identical appearance of treatments<br>Blind maintained until after trial concluded<br>Pain assessed 0.5, 1, 2, 3, 4, 5, 6, 7, 8 hours<br>Outcomes: standard 4 point PI scale, 5 point PR scale and 5 point patient global assessment scale, plus dysmenorrhoea symptom scorers	R = 2<br>DB = 2<br>W = 1<br>Total = 5/5<br><br>RoB size unclear	TOTPAR 6:<br>Cycle 1<br>DK 50 = 12.9<br>DK 100 = 14.0<br>Nap 550 = 12.9<br>Pbo = 7.4<br><br>Cycle 2<br>DK 50 = 13.0<br>DK 100 = 13.8<br>Nap 550 = 12.1<br>Pbo = 7.7	Cycle 1<br>DK 50 = 60/100<br>DK 100 = 67/101<br>Nap 550 = 55/92<br>Pbo = 27/90<br><br>Cycle 2<br>DK 50 = 54/89<br>DK 100 = 62/96<br>Nap 550 = 46/83<br>Pbo = 25/82	Very good or excellent<br>Cycle 1<br>DK 50 = 50/101<br>DK 100 =43/98<br>Nap 550 = 27/91<br>Pbo = 13/89<br><br>Cycle 2<br>DK 50 = 46/96<br>DK 100 = 46/89<br>Nap 550 = 30/83<br>Pbo = 21/82	Cycle 1<br>DK 50 = >8<br>DK 100 = >8<br>Nap 550 = >8<br>Pbo = >8<br><br>Cycle 2<br>DK50 = >8<br>DK100 = >8<br>Nap 550 = >8<br>Pbo = >8	By 8 hours<br>Cycle 1<br>DK 50 = 3/101<br>DK100 = 1/100<br>Nap 550 = 3/92<br>Pbo = 17/90<br><br>Cycle 2<br>DK 50 = 2/96<br>DK100 = 0/89<br>Nap 550 = 2/83<br>Pbo = 12/82	Not given	DK50 = 49/101<br>DK100 = 42/100<br>Nap 550 = 43/92<br>Pbo = 43/90	No withdrawals within first dose	No serious events evident in whole dosing schedule

The Oxford Quality score is based on randomisation (0, 1, or 2 points), double blinding (0, 1, or 2 points), and withdrawals (0 or 1 point). ASA, aspirin; CSR = clinical study report; DB = double blinding; DK = diclofenac potassium; Nap = naproxen; Pbo = placebo; R = randomisation; W = withdrawals.
